# Supplementary material for: NINJ1 induces plasma membrane rupture and release of damage-associated molecular pattern molecules during ferroptosis
Source: EMBO J. 2024 Feb 23;43(7):3. doi: 10.1038/s44318-024-00055-y (PMC10987646; doi:10.1038/s44318-024-00055-y)
Supplement: Supplementary file 1 — Appendix [file 44318_2024_55_MOESM1_ESM.pdf]

Appendix

NINJ1 induces plasma membrane rupture and release of damage-associated molecular pattern molecules during ferroptosis

Table of Contents

Appendix Figure S1. 2

NINJ1 deficiency blocks LDH release in ferroptotic fibroblast and BMDMs but only partially affects LDH release in RAW 264.7 cells 2

Appendix Figure S2. 3

NINJ1 controls plasma membrane permeabilization during ferroptosis in macrophages and fibroblasts 3

Appendix Figure S3. 5

NINJ1 oligomerization precedes plasma membrane permeabilization 5

Appendix Figure S4. 6

NINJ1 controls the release of DAMPs from ferroptotic iBMDMs 6

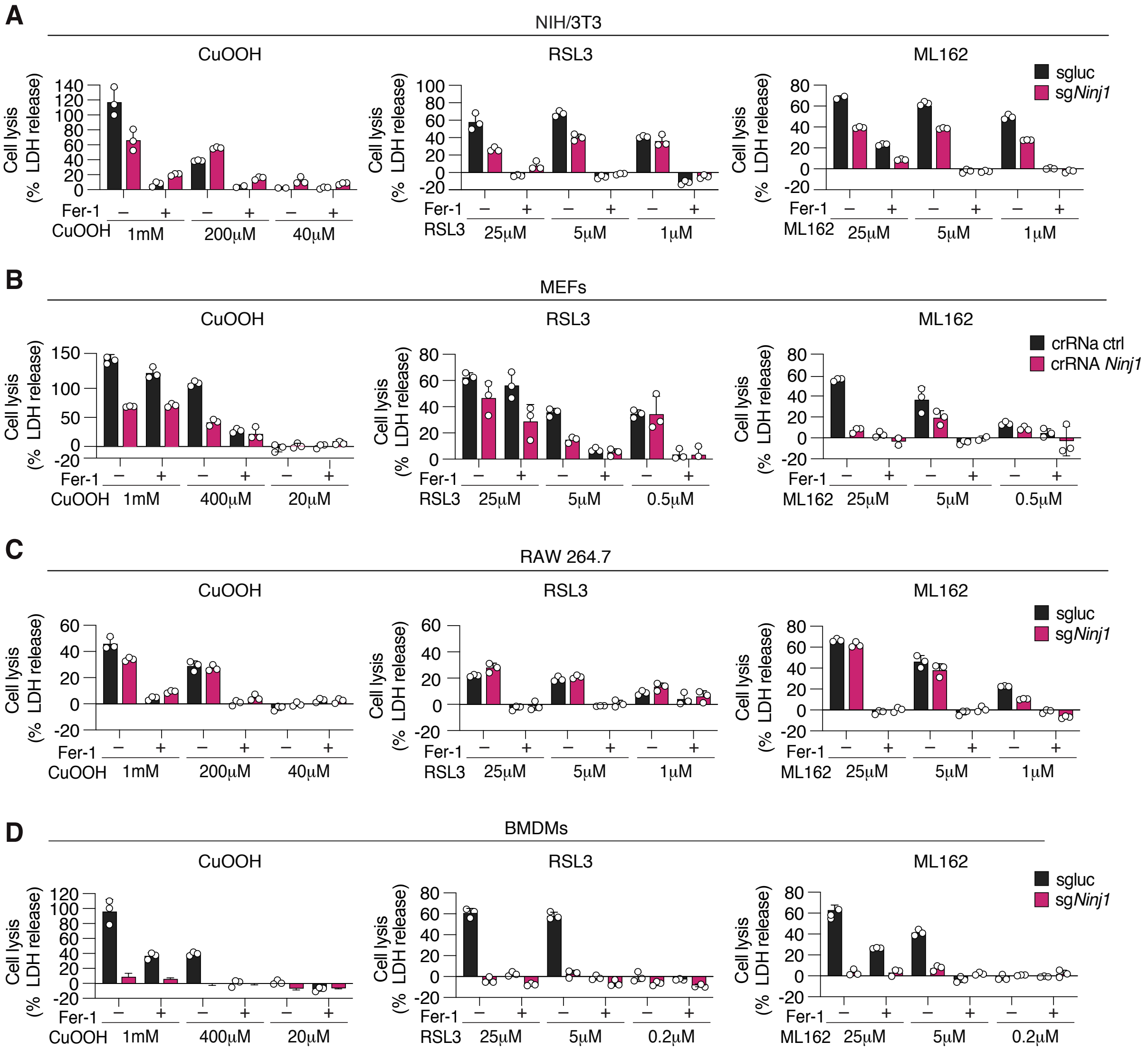

Appendix Figure S1. NINJ1 deficiency blocks LDH release in ferroptotic fibroblast and BMDMs but only partially affects LDH release in RAW 264.7 cells

A LDH release in WT and *Ninj1*$^{-/-}$ NIH/3T3 after treatment with different concentrations of CuOOH for 5 h, RSL3 for 8 h or ML162 for 8h.

B LDH release in WT and *Ninj1*$^{-/-}$ MEFs after treatment with different concentrations of CuOOH for 5 h, RSL3 for 8 h or ML162 for 8h.

C LDH release in WT and *Ninj1*$^{-/-}$ RAW 264.7 after treatment with different concentrations of CuOOH 1mM 2 h, RSL3 for 3 h or ML162 for 2 h

D LDH release in WT and *Ninj1*$^{-/-}$ BMDMs after treatment with different concentrations of CuOOH 5 h, RSL3 for 8 h or for 8 h

When indicated, 25μM Fer-1 was added simultaneously with ferroptosis activators (A-D).

Data information: All graphs show the mean ± SD. Data are representative from three independent experiments (A-D).

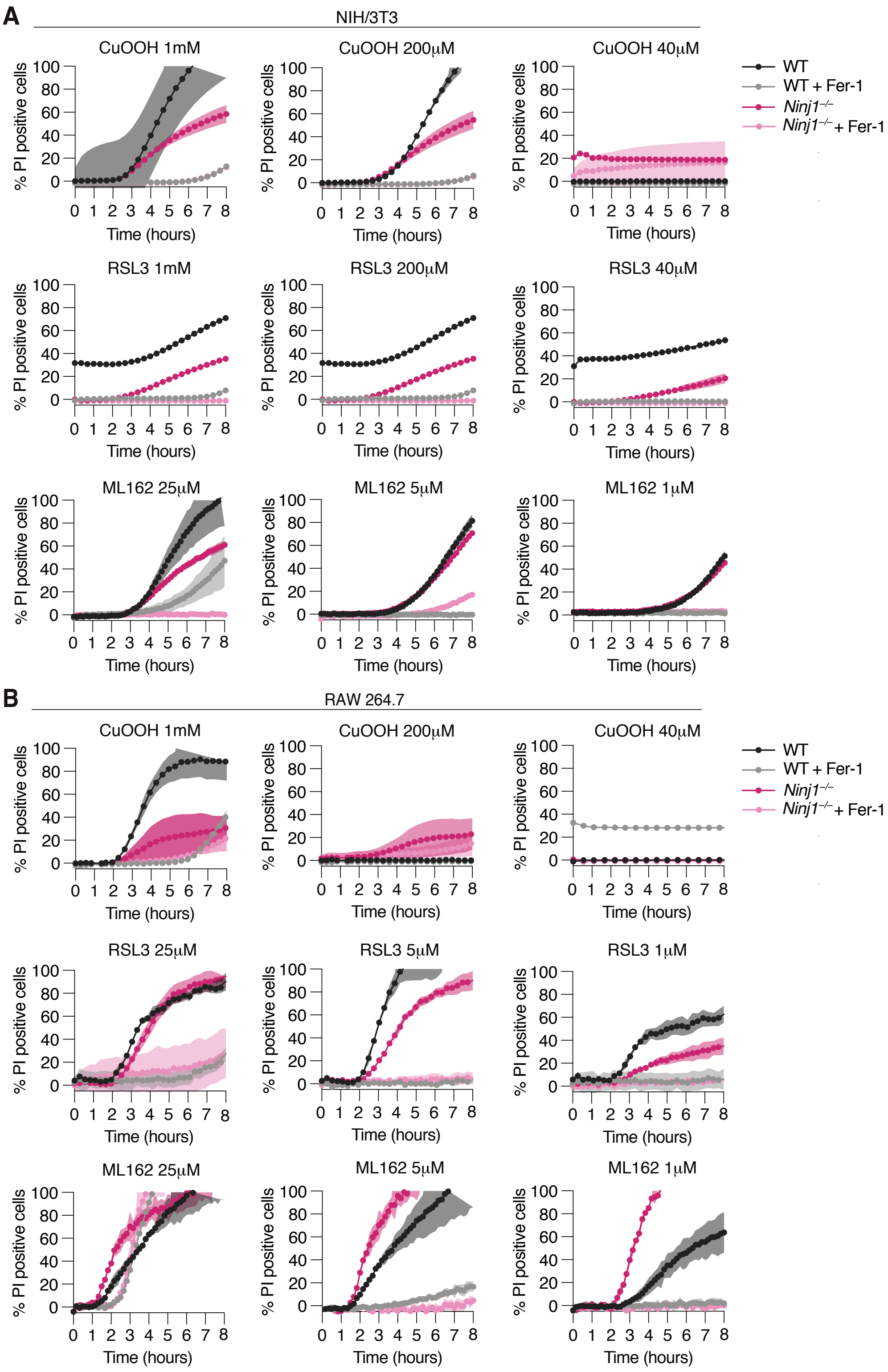

Appendix Figure S2. NINJ1 controls plasma membrane permeabilization during ferroptosis in macrophages and fibroblasts

A, B Percentage of propidium iodide (PI, Mw = 668 Da) uptake in WT and *Ninj1*$^{-/-}$ NIH/3T3 (A) and RAW 264.7 (B) over time (0-8 h) after treatment with different concentrations of

CuOOH, RSL3 or ML162. When indicated, 25μM (Fer-1) was added simultaneously with ferroptosis activators.

Data information: All graphs show the mean ± SD. Data are representative of three (A, B) independent experiments performed in triplicate.

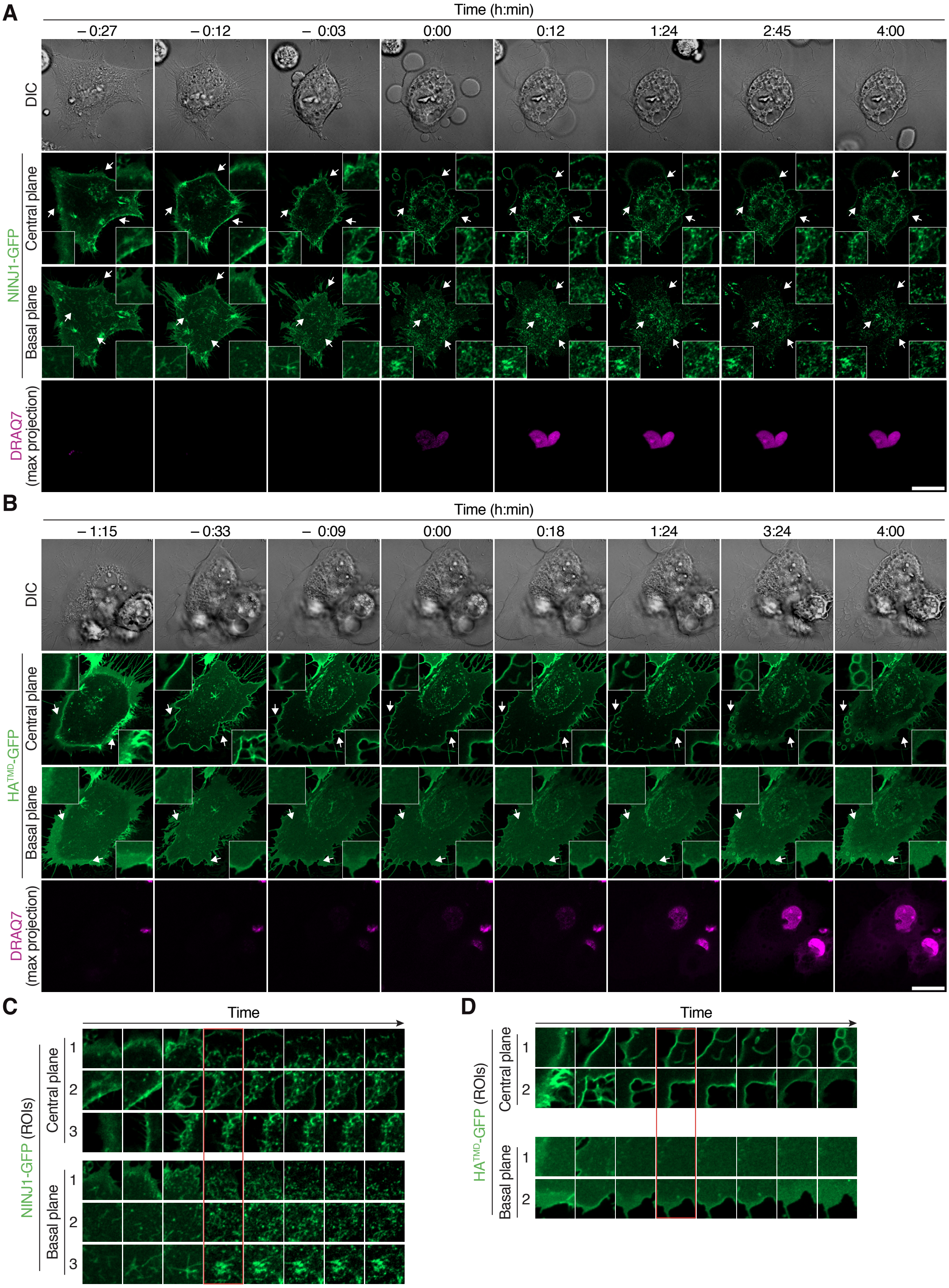

Appendix Figure S3. NINJ1 oligomerization precedes plasma membrane permeabilization

A-B Time-lapse fluorescence confocal microscopy of HeLa cells expressing hNINJ1-GFP(A) or HA^{TMD}-GFP (B) after CuOOH treatment. A) Same cell as in figure 5H. Images show the green fluorescence at the basal or central plane of the cells and the influx of DRAQ7 (maximum projection from a z-stack) to track plasma membrane permeabilization. White arrows point to regions enlarged in the insets. Time was normalized to the onset of increase in DRAQ7 nuclear fluorescence. Scale bars: 20 μm.

C-D Inset images from A (C) or B (D) showing NINJ1-GFP or HA^{TMD}-GFP distribution over time. Red squares highlight time zero, i.e. the onset of increase in DRAQ7 nuclear fluorescence.

Data information: Data are representative of 8 (A, C) or 6 (B, D) independent experiments.

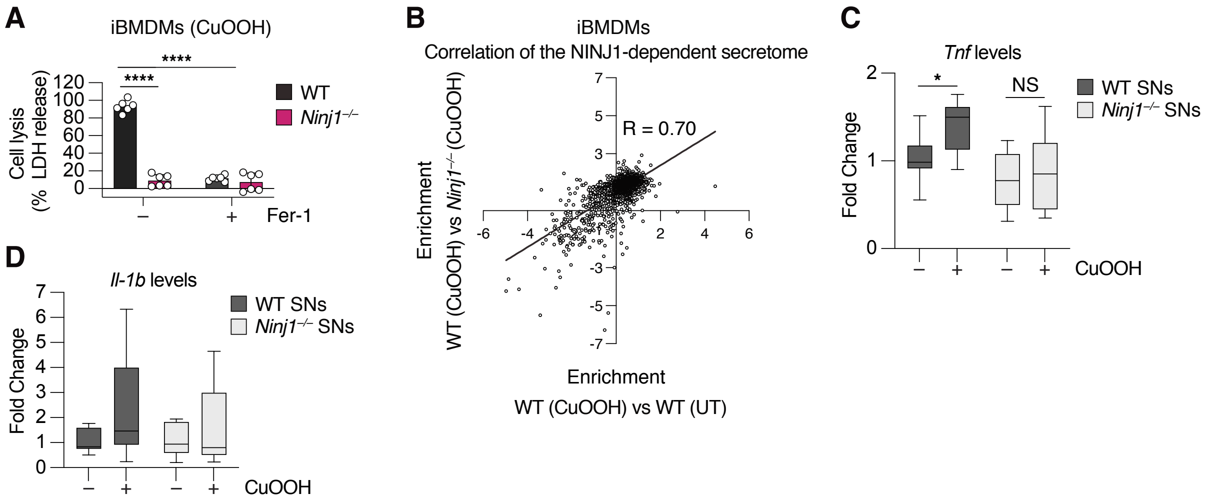

Appendix Figure S4. NINJ1 controls the release of DAMPs from ferroptotic iBMDMs

A LDH release in WT and *Ninj1*$^{-/-}$ iBMDMs treated with 1mM CuOOH for 3 h +/- 25µM Fer-1.

B Comparison of protein enrichments in WT (CuOOH) with respect to WT (UT) (x-axis) and WT (CuOOH) with *Ninj1*$^{-/-}$ (CuOOH) (y-axis) displays a correlation of R = 0.70. Each dot represents one protein.

C, D Fold change of mRNA expression of *Tnf* (E) and *Il1b* (F) in WT iBMDMs incubated for 16h with supernatants (SN) from WT or *Ninj1*$^{-/-}$ iBMDMs treated with 1mM CuOOH for 2 h.

Data information: Data are representative from two (A,B) independent experiments. Statistical analysis was done using unpaired t test with Welch's correction. ** $P < 0.1$ and ≥ 0.05 not shown.
