# Supplementary material for: NINJ1 induces plasma membrane rupture and release of damage-associated molecular pattern molecules during ferroptosis
Source: EMBO J. 2024 Feb 23;43(7):3. doi: 10.1038/s44318-024-00055-y (PMC10987646; doi:10.1038/s44318-024-00055-y)
Supplement: Supplementary file 3 — Movie EV1 [file 44318_2024_55_MOESM3_ESM.zip › 115042_Movie_EV1/Movie EV1.docx]

**Movie EV1**

Hela cells expressing NINJ1-GFP treated with 1mM CuOOH in the presence of 1μM DRAQ7, mebrane-impermeable DNA dye. Upon ferroptosis induction, NINJ1 was homogeneously distributed at the plasma membrane and latter it formed clusters at the plasma membrane, followed by DRAQ7 influx. NINJ1 (green) or DRAQ7 (purple). Images were acquired every 3 min. Same cell as in figure 5H and in Appendix Figure S3A.
