# Supplementary material for: NINJ1 induces plasma membrane rupture and release of damage-associated molecular pattern molecules during ferroptosis
Source: EMBO J. 2024 Feb 23;43(7):3. doi: 10.1038/s44318-024-00055-y (PMC10987646; doi:10.1038/s44318-024-00055-y)
Supplement: Supplementary file 4 — Movie EV2 [file 44318_2024_55_MOESM4_ESM.zip › 115042_Movie_EV2/Movie EV2.docx]

**Movie EV2**

Hela cells expressing the transmembrane domain of Hemagglutinin tagged with GFP (HATMD-GFP) treated with 1mM CuOOH in the presence of 1μM DRAQ7, mebrane-impermeable DNA dye. Upon ferroptosis induction, HATMD-GFP was homogeneously distributed at the plasma membrane and did not form clusters at the plasma membrane, we observed DRAQ7 influx. HATMD-GFP (green) or DRAQ7 (purple). Images were acquired every 3 min. Same cell as in Appendix Figure S3B.
