# Supplementary figures and images for: NINJ1 induces plasma membrane rupture and release of damage-associated molecular pattern molecules during ferroptosis
Source: EMBO J. 2024 Feb 23;43(7):3. doi: 10.1038/s44318-024-00055-y (PMC10987646; doi:10.1038/s44318-024-00055-y)

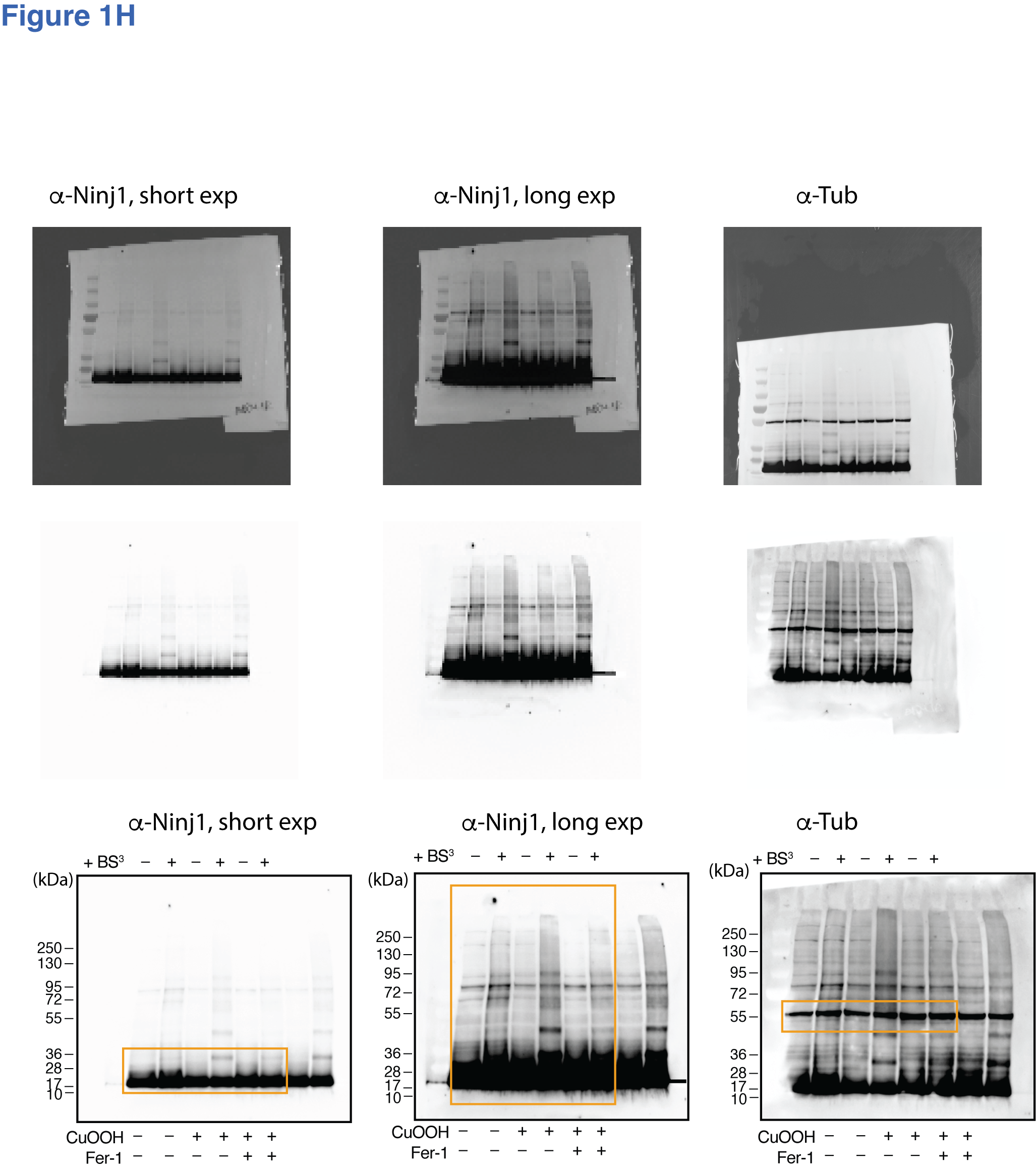

Supplement: Supplementary file 5 — Source Data Fig. 1 [file 44318_2024_55_MOESM5_ESM.zip › Figure_1_2024/1H/Image_data_fig1H_WB_CuOOH_2024.tif]

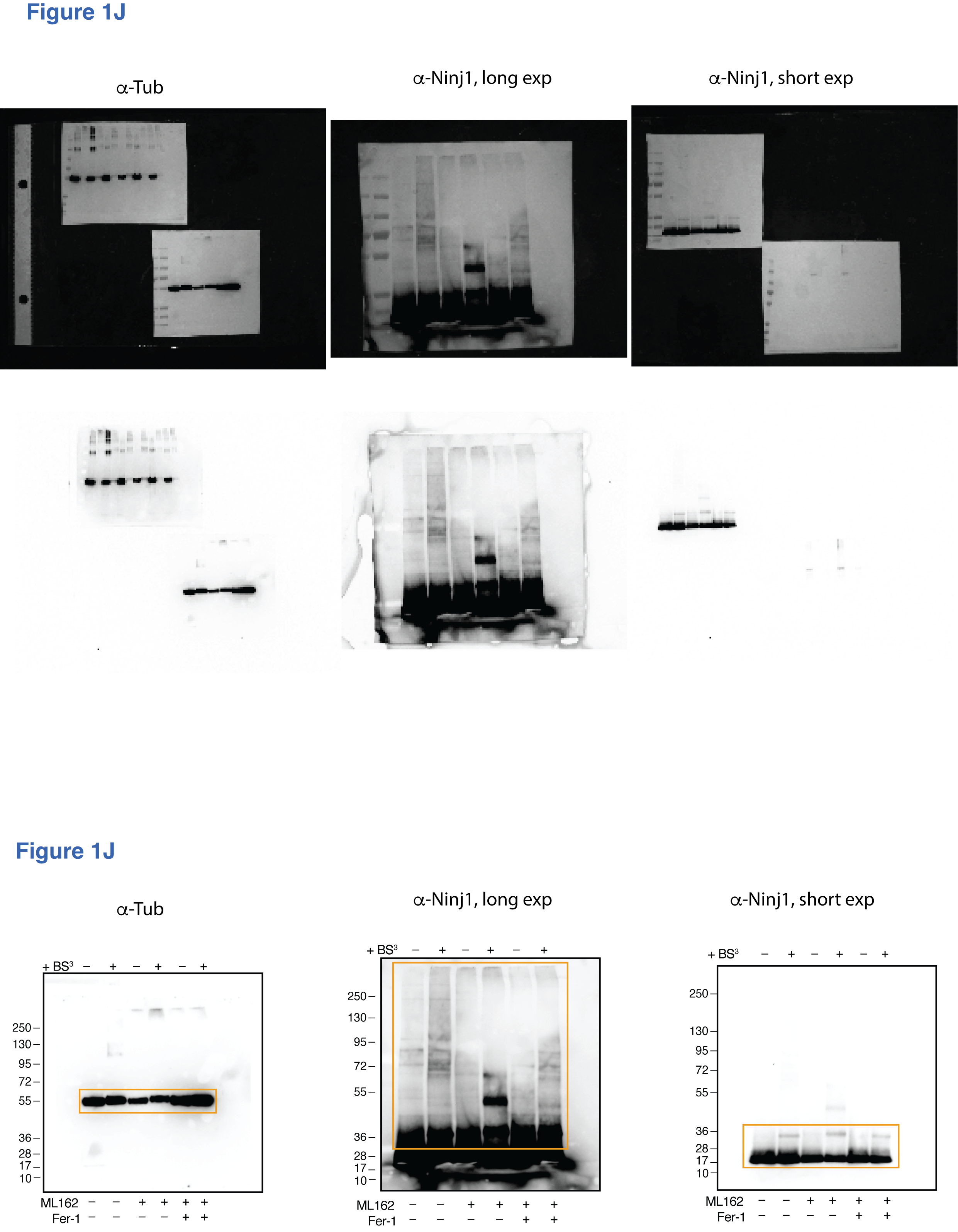

Supplement: Supplementary file 5 — Source Data Fig. 1 [file 44318_2024_55_MOESM5_ESM.zip › Figure_1_2024/1J/Image_data_fig1_J_WB_ML162_2024.tif]

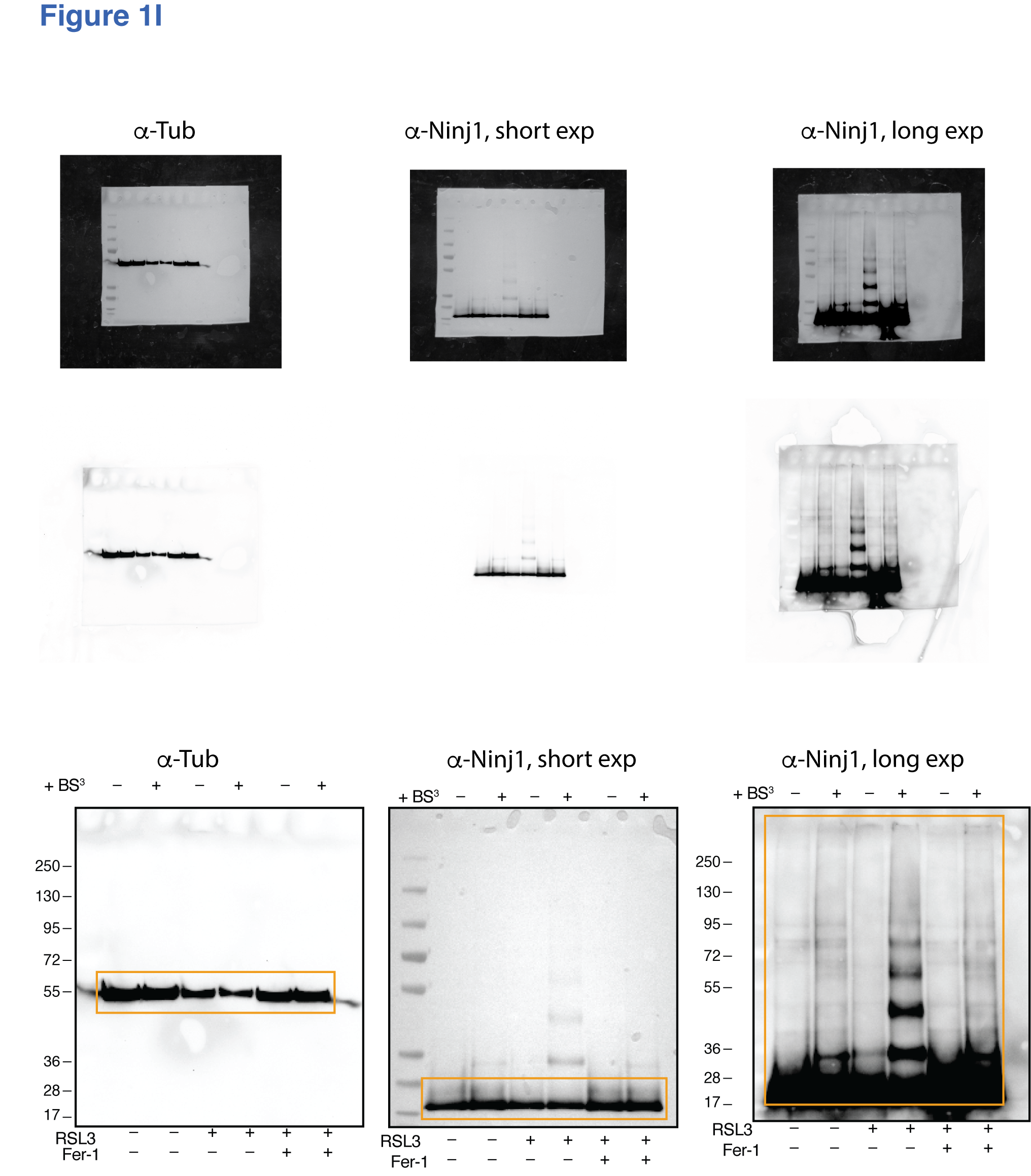

Supplement: Supplementary file 5 — Source Data Fig. 1 [file 44318_2024_55_MOESM5_ESM.zip › Figure_1_2024/1I/Image_data_fig1_I_WB_RSL3_2024.tif]

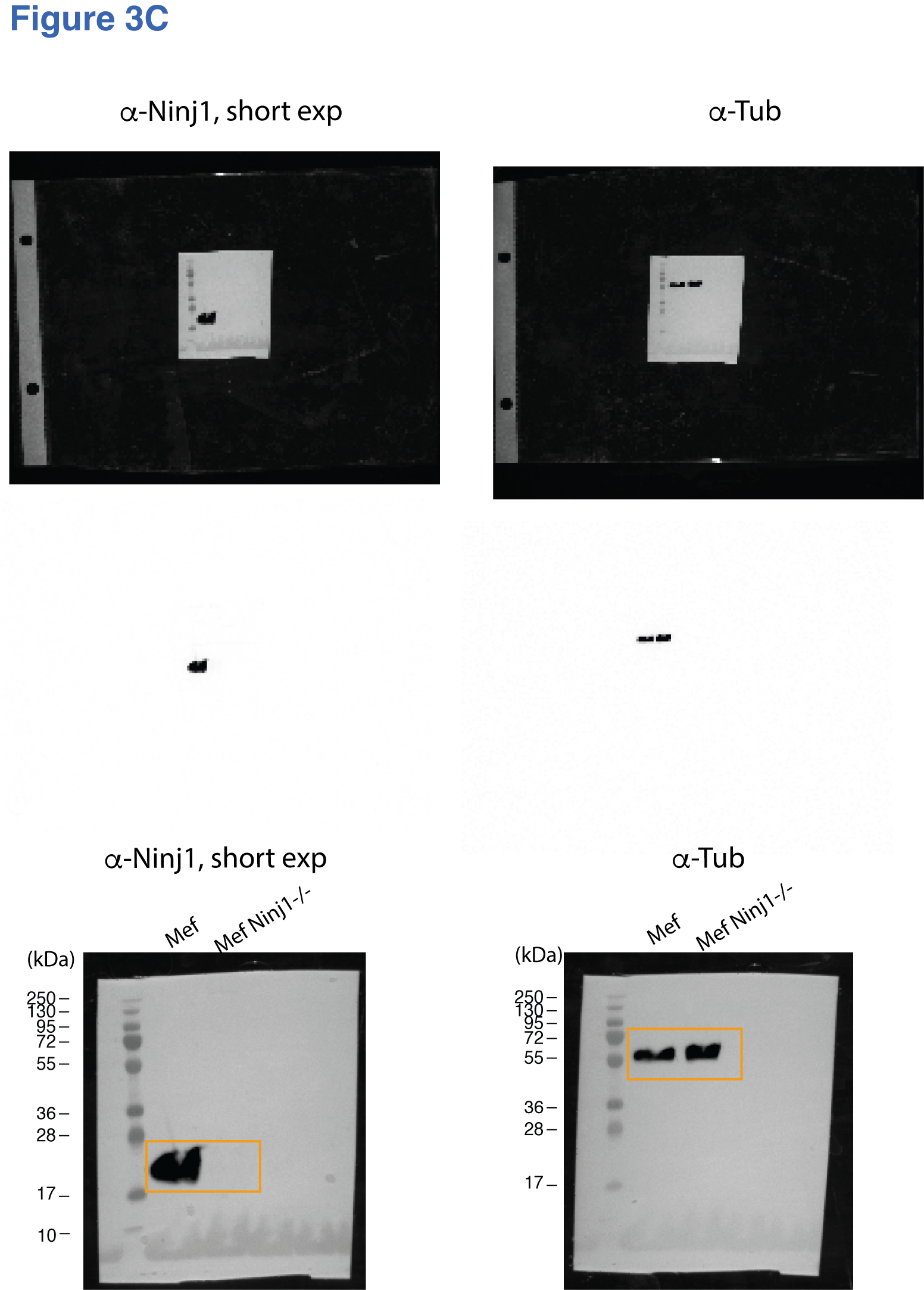

Supplement: Supplementary file 7 — Source Data Fig. 3 [file 44318_2024_55_MOESM7_ESM.zip › Figure_3_2024/3C/Image_data_fig3C_WB_MEFs_2024.tif]

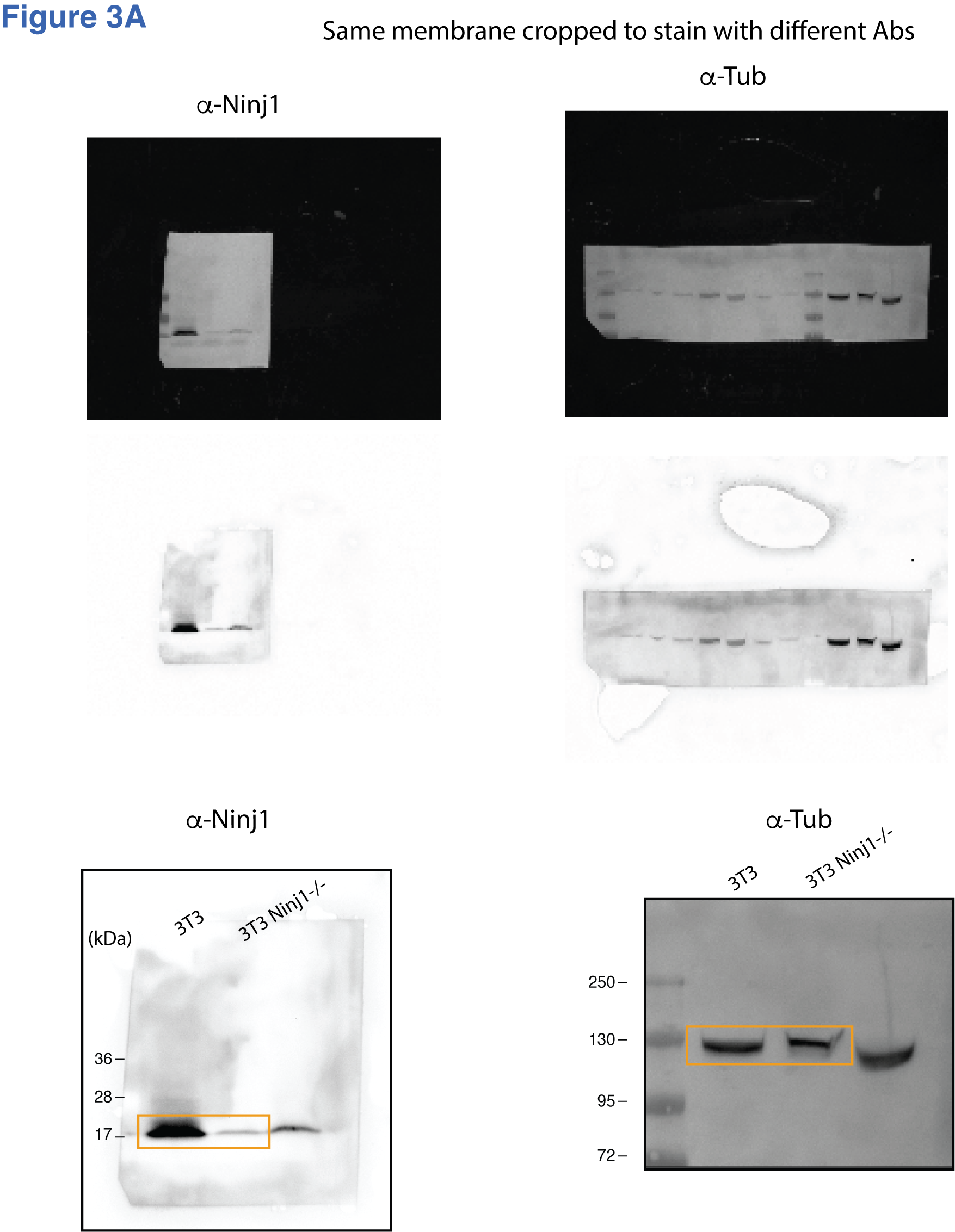

Supplement: Supplementary file 7 — Source Data Fig. 3 [file 44318_2024_55_MOESM7_ESM.zip › Figure_3_2024/3A/Image_data_fig3A.WB_3T3_2024.tif]

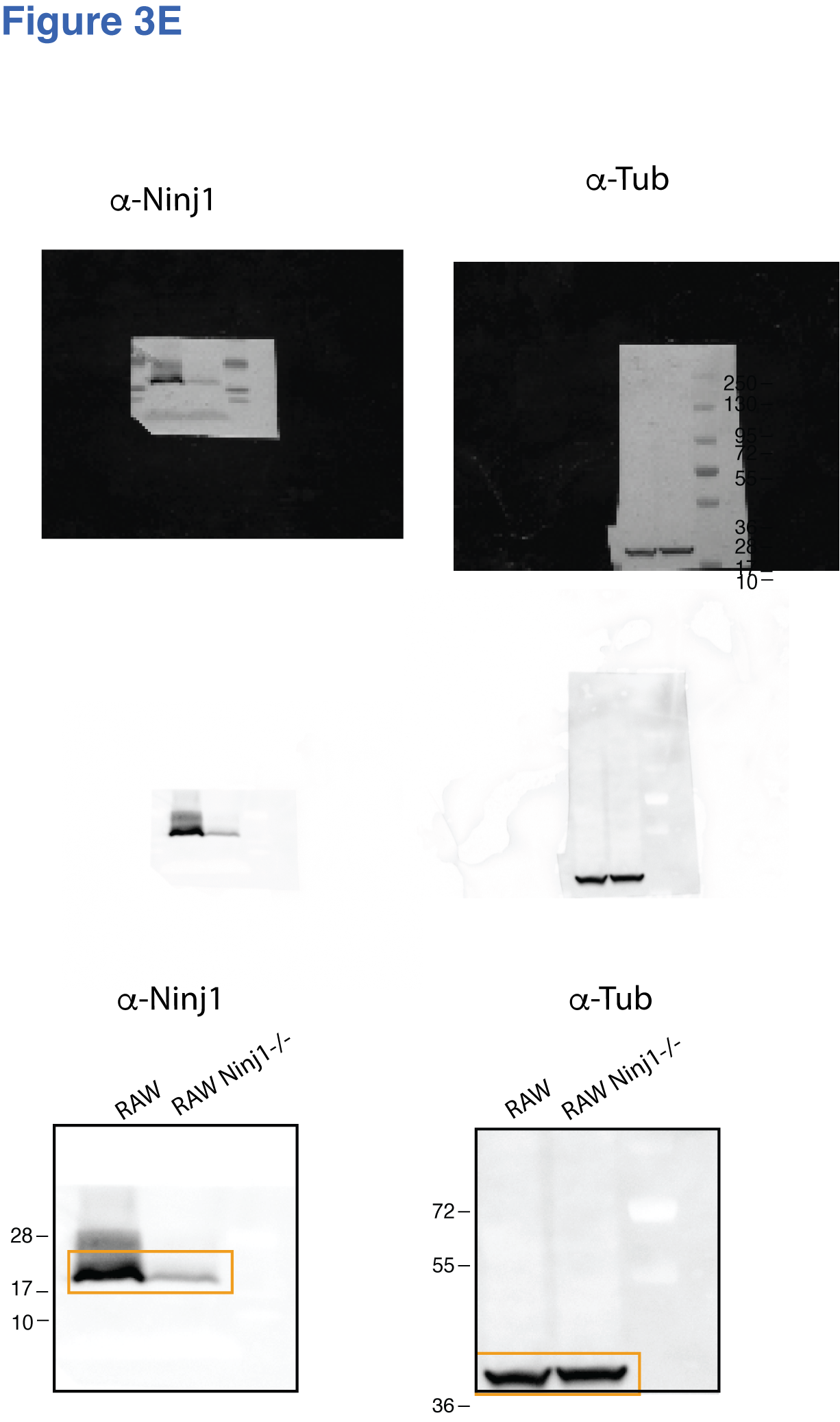

Supplement: Supplementary file 7 — Source Data Fig. 3 [file 44318_2024_55_MOESM7_ESM.zip › Figure_3_2024/3E/Image_data_fig3E_WB_2024.tif]
