# Supplementary material for: NINJ1 induces plasma membrane rupture and release of damage-associated molecular pattern molecules during ferroptosis
Source: EMBO J. 2024 Feb 23;43(7):3. doi: 10.1038/s44318-024-00055-y (PMC10987646; doi:10.1038/s44318-024-00055-y)
Supplement: Supplementary file 11 — Expanded View Figures [file 44318_2024_55_MOESM11_ESM.pdf]

Expanded View Figures

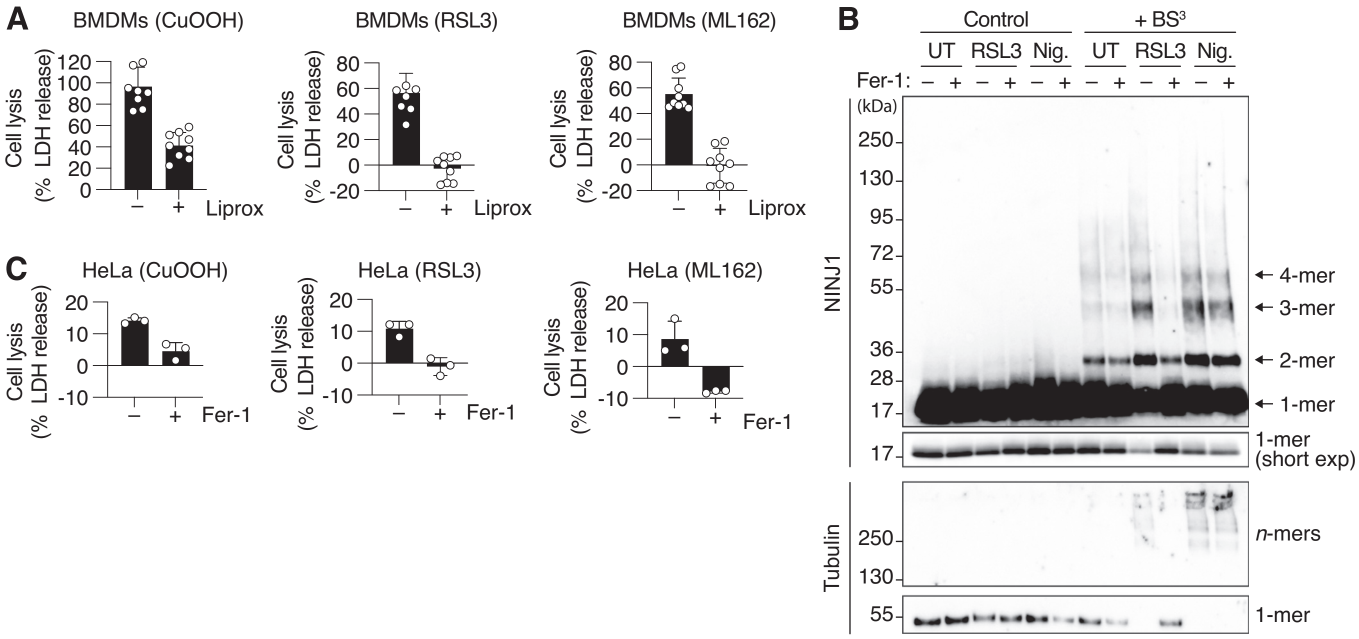

Figure EV1. Ferroptosis inhibitors specifically block ferroptosis in BMDMs.

(**A**) LDH release in WT BMDMs treatment with 1 mM CuOOH for 5 h, 5 μM RSL3 for 6 h, or 5 μM ML162 for 5 h. When indicated, 1 μM Liprox was added simultaneously with CuOOH, RSL3, or ML162. Liprox = Liproxstatin-1. (**B**) Western blot analysis of endogenous NINJ1 in WT BMDMs treated with 5μg/mL nigericin for 1.5 h or 5 μM RSL3 for 4 h followed by treatment with the membrane-impermeable crosslinker BS^3. Mixed supernatant and cell extracts were analyzed. FL, full length; Short exp = short exposure; 1-mer = monomer; 2-mer = dimer; 3-mer = trimer; 4-mer = tetramer and n-mer, higher-order oligomers. (**C**) LDH release in HeLa cells after treatment with 1 mM CuOOH for 3 h, 5 μM RSL3 for 3 h, or 5 μM ML162 for 3 h. When indicated, 25 μM Fer-1 was added simultaneously with ferroptosis and pyroptosis activators (**B**, **C**). Data information: All graphs show the mean ± SD. Data are pooled from three independent experiments (**A**) or representative of two (**B**, **C**) independent experiments performed in triplicate.

 © The Author(s)

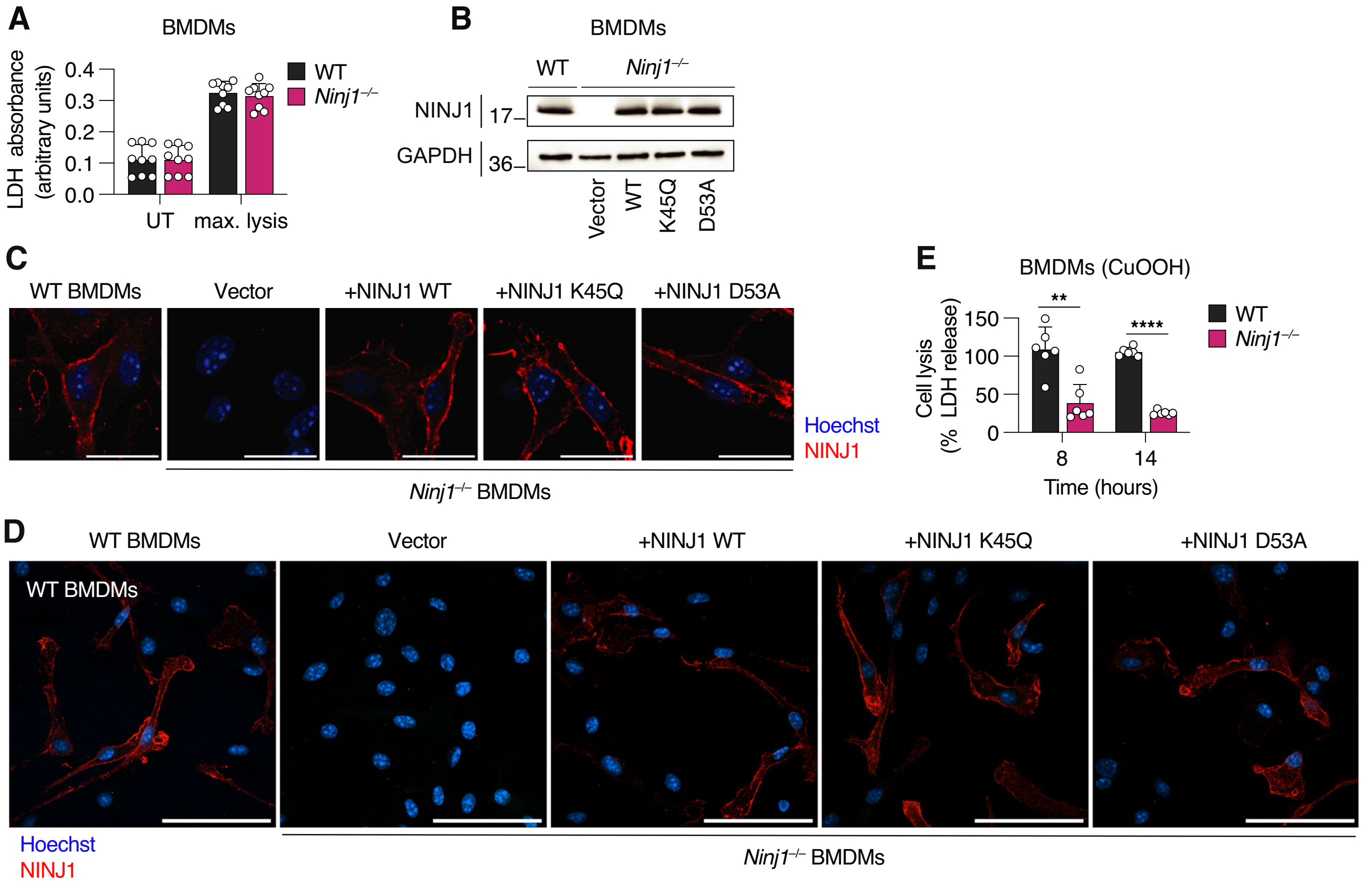

Figure EV2. NINJ1 filament formation is essential to induce cell lysis in ferroptosis.

(**A**) LDH absorbance in WT and *Ninj1*$^{-/-}$ BMDMs untreated (UT) or lysed with Triton X-100 to a final concentration of 0.01% (max. lysis). (**B**) Western blot of NINJ1 expression in WT or *Ninj1*$^{-/-}$ BMDMs transduced with a retroviral vector expressing WT mNINJ1 or different mNINJ1 mutants. Transduction with a GFP expressing vector was used as a control. Cell extracts were analyzed. GAPDH is a loading control. (**C**, **D**) Immunofluorescence confocal microscopy of NINJ1 (red) in WT or *Ninj1*$^{-/-}$ BMDMs complemented with WT or different NINJ1 mutants upon retroviral transduction. Scale bars: 20 µm (**C**) and 60 µm (**D**). (**E**) LDH release in WT and *Ninj1*$^{-/-}$ BMDMs upon treatment with 1 mM CuOOH for 8 or 14 h. Data information: All graphs show the mean ± SD. Data are pooled from three independent experiments performed in triplicate (**A**, **E**) or representative of two different experiments (**B**, **C**, **D**). Statistical analysis was done using Student's unpaired two-sided t-test. **** $P < 0.0001$, ** < 0.01.

© The Author(s)

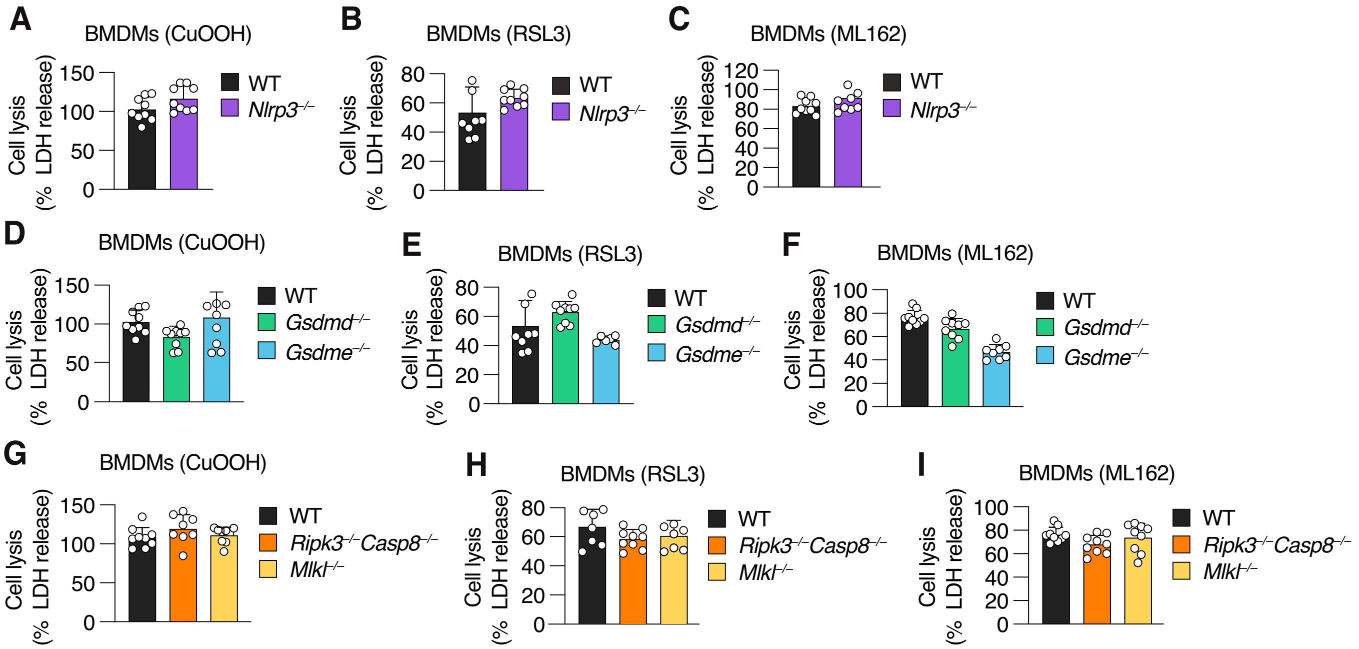

Figure EV3. NINJ1 is the only driver of cell lysis during ferroptosis.

(**A–I**) LDH release in WT and *Nlrp3*$^{-/-}$ BMDMs treated with 1 mM CuOOH for 5 h (**A**), 5 μM RSL3 for 6 h (**B**), or 5 μM ML162 for 5 h (**C**), WT, *Gsdmd*$^{-/-}$, and *Gsdme*$^{-/-}$ BMDMs treated with 1 mM CuOOH for 5 h (**D**), 5 μM RSL3 for 6 h (**E**) or 5 μM ML162 for 5 h (**F**) and WT, *Ripk3*$^{-/-}$/*Casp8*$^{-/-}$, and *Mlkl*$^{-/-}$ treated with 1 mM CuOOH for 5 h (**G**), 5 μM RSL3 for 6 h (**H**), or 5 μM ML162 for 5 h (**I**). Data information: All graphs show the mean ± SD. Data are pooled from three independent experiments performed in triplicate (**A–I**). Statistical analysis was done using Student's unpaired two-sided t-test (**A–C**) or one-way ANOVA (**D–I**).

 © The Author(s)

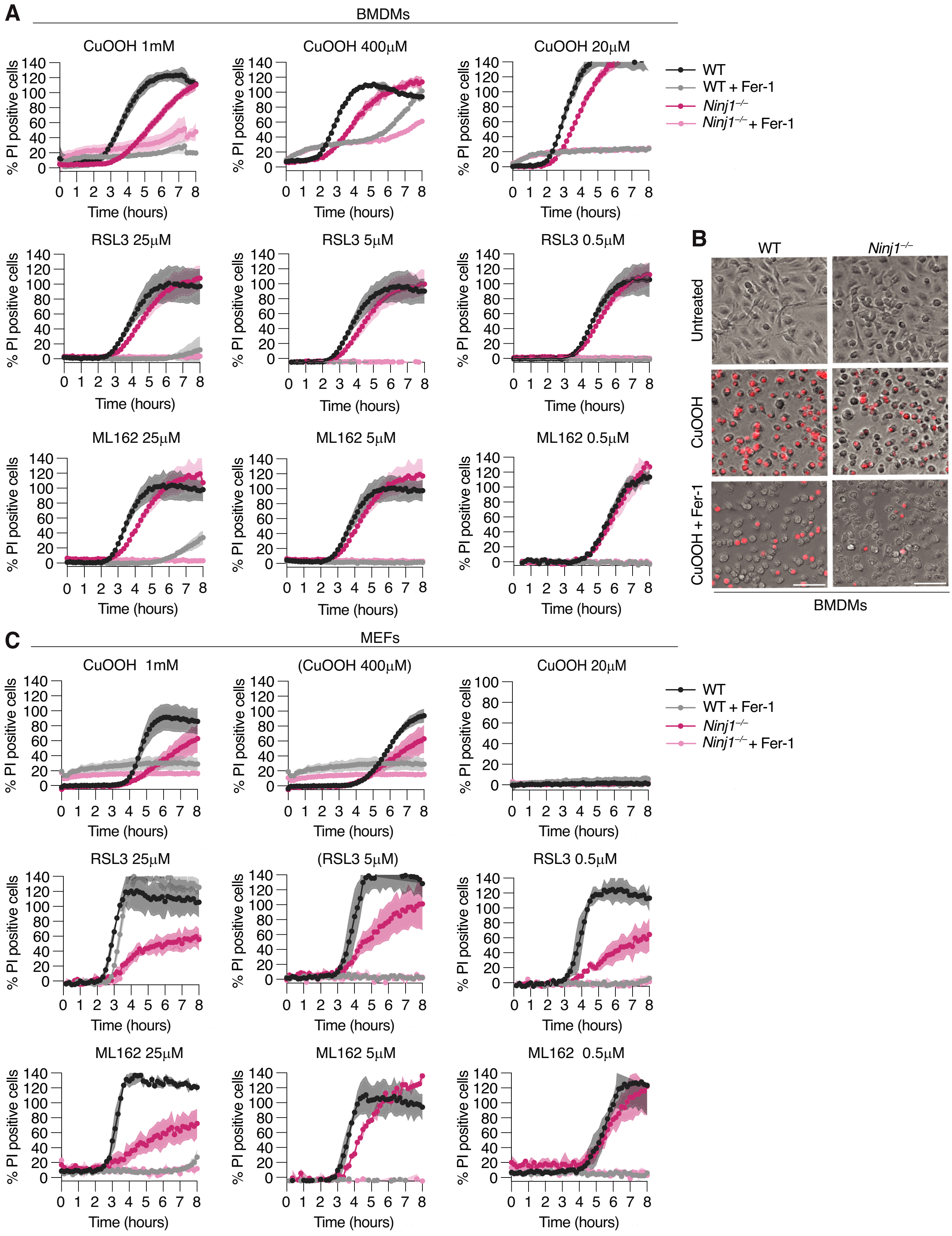
A
BMDMs
CuOOH 1mM
CuOOH 400μM
CuOOH 20μM
WT
WT + Fer-1
Ninj1−/−
Ninj1−/− + Fer-1
RSL3 25μM
RSL3 5μM
RSL3 0.5μM
ML162 25μM
ML162 5μM
ML162 0.5μM
% PI positive cells
Time (hours)
B
WT
Ninj1−/−
Untreated
CuOOH
CuOOH + Fer-1
BMDMs
C
MEFs
CuOOH 1mM
(CuOOH 400μM)
CuOOH 20μM
RSL3 25μM
(RSL3 5μM)
RSL3 0.5μM
ML162 25μM
ML162 5μM
ML162 0.5μM

© The Author(s)

Figure EV4. NINJ1 controls plasma membrane permeabilization during ferroptosis in macrophages and fibroblasts.

(**A**) Percentage of propidium iodide (PI, Mw = 668 Da) uptake in WT and *Ninj1*$^{-/-}$ BMDMs over time (0–8 h) after treatment with different concentrations of CuOOH, RSL3, or ML162. (**B**) Representative images showing PI uptake (red) in WT and *Ninj1*$^{-/-}$ BMDMs left untreated or after treatment with 1 mM CuOOH for 5 h. Scale bar: 200 μm. (**C**) Percentage of propidium iodide (PI, Mw = 668 Da) uptake in WT and *Ninj1*$^{-/-}$ MEFs over time (0–8 h) after treatment with different concentrations of CuOOH, RSL3, or ML162. When indicated, 25 μM Fer-1 was added simultaneously with ferroptosis activators (**A–C**). Data information: All graphs show the mean ± SD. Data are representative of three (**A**, **C**) or two (**B**) independent experiments performed in triplicate.

 © The Author(s)

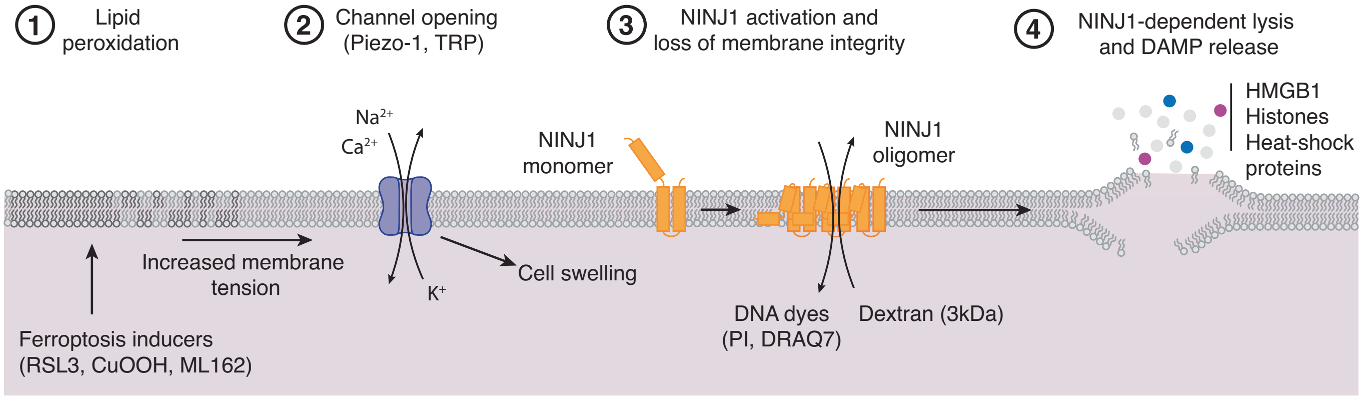

Figure EV5. Model for ferroptosis-associated cell lysis.

(1) Ferroptosis inducers promote the formation of lipid peroxides at the plasma membrane which cause and increase in membrane tension. (2) Mechanosensitive ion channels (Piezo-1 and TRP channels) open is response to increased membrane tension, resulting in the entry of calcium and sodium ions and the release of potassium ions. These non-selective ion fluxes cause an increase in the cell volume and swelling (3) Following this step, NINJ1 is activated, possibly because of ion disbalance, swelling or a yet unrecognized signal, and oligomerizes to form amphipathic filaments. Initially these filaments cause only small lesions that allow the entry and exit of small molecules, such as DNA-binding dyes. (4) Eventually NINJ1 lesions cause complete membrane rupture and cell lysis. As a consequence of NINJ1-dependent PMR, ferroptotic cells start to leak intracellular content among them many DAMPs that can drive inflammation.
